# Biomechanics and Bioengineering of Orthopedic and Cardiovascular Rehabilitation

**DOI:** 10.1155/2018/1716809

**Published:** 2018-08-19

**Authors:** Lizhen Wang, Chia-Ying Lin, Wei Yao, Duo Wai-Chi Wong, Rui Zhu

**Affiliations:** ^1^Key Laboratory for Biomechanics and Mechanobiology of Ministry of Education, School of Biological Science and Medical Engineering, Beijing Advanced Innovation Centre for Biomedical Engineering, Beihang University, Beijing, China; ^2^Department of Biomedical, Chemical & Environmental Engineering, University of Cincinnati, Cincinnati, OH, USA; ^3^Department of Orthopaedic Surgery, University of Cincinnati, Cincinnati, OH, USA; ^4^Department of Neurosurgery, University of Cincinnati, Cincinnati, OH, USA; ^5^Department of Biomedical Engineering, University of Strathclyde, Glasgow, UK; ^6^Department of Biomedical Engineering, Faculty of Engineering, The Hong Kong Polytechnic University, Kowloon, Hong Kong, China; ^7^Spine Division of Orthopaedic Department, Tongji Hospital, Tongji University School of Medicine, Shanghai, China

Musculoskeletal problem, such as traumatic injury, osteoporosis, and osteoarthritis, is one of the leading causes of disability worldwide and is therefore a crucial branch of public health. Biomechanics has been proven to play a critical role in musculoskeletal pathology, treatment, and rehabilitation. Rehabilitation describes specialized healthcare dedicated to improving, maintaining, or restoring physiological strength, cognition, and mobility with maximized results. It helps an individual achieve the highest level of function or greatest independence and quality of life possible after illness, injury, or surgery using physical training, instruments, devices, or other methods. Advances in rehabilitation, including the theory, paradigm, protocol, modality, invention, and engineering, can improve the development of modern medicine and also the quality of life especially for the disabled and elderly people. The main focus of this special issue is on innovative theory and application of biomechanics to understand musculoskeletal pathology and to improve the techniques for the treatment and rehabilitation.

As the variety of topics presented in this special issue suggests, there are still many difficulties related to the mechanism of human injury and its rehabilitation and invention or development of the rehabilitation device. M. Zhang et al. explored the effects of scan resolutions and element sizes on QCT/FEA outcomes. They showed scan resolution and element size should be chosen optimally to improve the accuracy of QCT/FEA. To characterize treadmill exercise, N. Guo et al. calculated muscle forces in full gait cycle by inverse dynamic analysis. They found the constraint loading mode had an impact on the muscle forces in treadmill exercise, thus could be manipulated to enhance the effect of the muscle training in spaceflight. J. Gao et al. concluded that effects of treadmill exercise on joints may be associated with different additional weight-bearing levels, and exercise intensities during joint growth and maturation should be selected reasonably. Some new cutting-edge technologies such as robotics, wear devices, and Artificial Intelligence have been applied for the treatment and rehabilitation. S. J. Biggar et al. have developed and evaluated a wearable robotic glove for hand rehabilitation. Regarding the effect of design parameters of personalized orthopedic insole, S. Su et al. showed insole material and support design is positively affecting the correction of orthopedic insole, but negatively resulting unreasonable stress on the stress in the joint and ligaments.

Rehabilitation to stroke patients has been constantly receiving close review. Two research teams came up with cutting edge rehabilitation devices for stroke patients to regain, respectively, upper and lower limb motor functions. Q. Miao and colleagues designed a compact and user-friendly robotic exoskeleton for upper limb rehabilitation, which featured a real-time kinematic and dynamic-based system, and allowed both passive and interactive training. Their study demonstrated satisfactory positioning as well as force tracking performance and will head for field study for poststroke patients. Besides, X. Zeng et al. integrated the motion intention of stroke patients via steady-state visual evoked potential with virtual reality technology to develop a rehabilitation robot for the ankle joint. The motion intention-directed passive training was conducted on five subjects with a high success rate. The strategy enabled active engagement for subjects without the ability to conduct active training.

The complex interactions between neuromuscular prefatigue and brain control were investigated by L. Wang and colleagues. The research team induced anatagonist muscle prefatigue by isometric elbow flexion. The influence of the fatigue on the cortico-cortical coupling and central modulation was assessed though the technique of EEG-EEG phase synchronization index. Their findings revealed an enhanced intracortical signal which could be an indication for the compensation for the prefatigued-induced joint instability. On the other contrary, Y. Men et al. concerned the microdefect behavior of articular cartilage which implicates the problem of osteoarthritis. Precisely, Y. Men et al. applied a rolling load on porcine articular cartilage and observed the strain of the samples. They identified that the shear strain could be the main factor in cartilage destruction, while rolling velocity also played an important role on the destruction of the superficial and middle layers.

Furthermore, recent criteria for orthopedic and cardiovascular rehabilitation affect not only the diagnosis but also the treatment and possibly involves the innovation of supportive or protective devices. This is the reason why, not only will future research focus on mechanism and criteria but also it will increasingly include therapies with rehabilitation and its effects.

*Lizhen Wang* *Chia-Ying Lin* *Wei Yao* *Duo Wai-Chi Wong* *Rui Zhu*

